# Carfentanil Outbreak — Florida, 2016–2017

**DOI:** 10.15585/mmwr.mm6905a2

**Published:** 2020-02-07

**Authors:** Chris Delcher, Yanning Wang, Russell S. Vega, John Halpin, R. Matthew Gladden, Julie K. O’Donnell, Jessica A. Hvozdovich, Bruce A. Goldberger

**Affiliations:** ^1^Department of Pharmacy Practice and Science, University of Kentucky, Lexington; ^2^Department of Health Outcomes and Biomedical Informatics, University of Florida, Gainesville; ^3^District Twelve Medical Examiner’s Office, Sarasota, Florida; ^4^Division of Unintentional Injury Prevention, National Center for Injury Prevention and Control, CDC; ^5^Forensic Medicine Division, Department of Pathology, Immunology and Laboratory Medicine, College of Medicine, University of Florida, Gainesville.

Increased prevalence of illicitly manufactured fentanyl and fentanyl analogs has contributed substantially to overdose deaths in the United States (*1*–*3*). On October 26, 2015, CDC issued a Health Advisory regarding rapid increases in deaths involving fentanyl. This CDC Health Advisory has been updated twice to address increases in fentanyl and fentanyl analog overdoses and their co-occurrence with nonopioids (*4*). Deaths involving carfentanil, an analog reportedly 10,000 times more potent than morphine and 100 times more potent than fentanyl, were first reported in Florida, Michigan, and Ohio in 2016 and described in an August 2016 CDC Health Advisory (*1*,*5*). Carfentanil is used to rapidly immobilize large animals in veterinary medicine and has no U.S. approved therapeutic use in humans. Carfentanil’s street price per dose is likely lower than that of heroin. During 2016 and 2017, an outbreak of carfentanil-involved fatal overdoses in Florida emerged, and the Medical Examiner jurisdiction serving Sarasota, Manatee, and DeSoto counties (the Sarasota area) was the outbreak epicenter. This report describes toxicology profiles, sociodemographic information, and geographic distributions of carfentanil-involved fatal overdoses (carfentanil deaths) in the Sarasota area compared with those in the rest of Florida (i.e., all Florida counties excluding Sarasota area) from January 2016 to December 2017. The Sarasota area accounted for 19.0% of 1,181 statewide carfentanil deaths that occurred during this time and experienced a peak in carfentanil deaths preceding the larger Florida outbreak. The report of a single carfentanil death from August to December 2017 (compared with 73 reported deaths during the same period in 2016) appeared to mark the end of the outbreak in the area. The threat of such rapid, intense fatal overdose outbreaks highlights the need for accelerated reporting, reliable data sharing systems, and novel proactive surveillance to support targeted prevention and response efforts by public health and safety organizations (*6*).

Florida medical examiners report drug-related deaths to the Florida Department of Law Enforcement with details including cause and manner of death, demographic data, and toxicology findings. In Florida, fentanyl analog reporting (including carfentanil) began in January 2016.* Deaths were determined by medical examiners as fentanyl analog–caused deaths, and carfentanil was listed in the toxicology report. A substance was considered to be co-occurring if it was detected by toxicologic testing irrespective of whether the medical examiner determined that it contributed to the fatal overdose. Descriptive statistics, epidemic curves, and maps were used to describe the carfentanil outbreak and standard distribution tests (statistical significance defined as p<0.05) were used to compare characteristics of the Sarasota area outbreak with those of the outbreak in the rest of Florida. SAS (version 9.4; SAS Institute) was used to conduct all analyses.

Florida experienced 1,181 carfentanil-involved overdose deaths from 2016 (548) to 2017 (633). Among these, 224 (19.0%) occurred in the Sarasota area (Table), although according to the U.S. Census Bureau, this region accounts for only 4.0% of Florida’s population.^†^ The Sarasota area outbreak began with four carfentanil deaths in June 2016, and peaked at 37 deaths in July, accounting for 82.2% of the area’s opioid overdose deaths that month (Figure 1) and 50% of all opioid overdose deaths in 2016. Carfentanil deaths in the Sarasota area declined substantially by the end of 2016 but increased again in January 2017 and remained elevated through July 2017 (110 deaths, 54% of all 2017 opioid overdose deaths). Only one death was reported during August–December 2017. The Sarasota area had the highest rate of carfentanil deaths for 2016 (13.8 per 100,000) and the second highest rate for 2017 (13.1 per 100,000), following the Palm Beach area (19.4 per 100,000) (Figure 2). In the rest of Florida, the number of carfentanil deaths peaked in October 2016, approximately 3 months after the initial Sarasota area spike, at 137 deaths (32.0% of Florida’s October opioid overdose deaths). Carfentanil deaths accounted for 12% and 13% of opioid deaths in the rest of Florida during 2016 and 2017, respectively. Among Florida’s 67 counties, 26 (39%) and 36 (54%) reported one or more carfentanil deaths in 2016 and 2017, respectively (Figure 2) (Supplementary Table, https://stacks.cdc.gov/view/cdc/84586), suggesting that the epidemic expanded from Florida’s southern to northwestern counties. After the Sarasota outbreak subsided, carfentanil was still consistently present in approximately 8% of state opioid deaths from August to December 2017 (Figure 1).

**TABLE:** Characteristics of carfentanil-involved overdose deaths — Sarasota area (Sarasota, Manatee, and DeSoto counties) and the rest of Florida, 2016 and 2017

Characteristic	2016 (N = 548)			2017 (N = 633)		
	Sarasota area n = 114	Rest of Florida n = 434	p-value*	Sarasota area n = 110	Rest of Florida n = 523	p-value*
	No. (%)	No. (%)		No. (%)	No. (%)	
**Age group (yrs)**						
<25	11 (9.7)	46 (10.6)	NS	9 (8.2)	64 (12.2)	NS
25–34	40 (35.1)	168 (38.7)		39 (35.4)	202 (38.6)	
35–44	33 (28.9)	107 (24.7)		31 (28.2)	123 (23.5)	
45–54	18 (15.8)	66 (15.2)		22 (20.0)	82 (15.7)	
≥55	12 (10.5)	47 (10.8)		9 (8.2)	52 (9.9)	
Median age (yrs)	35.5	35.0	—	36.0	34.0	—
**Sex**						
Female	22 (19.3)	94 (21.7)	NS	35 (31.8)	116 (22.2)	<0.05
Male	92 (80.7)	340 (78.3)		75 (68.2)	407 (77.8)	
**Race**						
White	105 (92.1)	391 (90.1)	NS	96 (87.3)	477 (91.2)	NS
Other	9 (7.9)	43 (9.9)		14 (12.7)	46 (8.8)	
**Co-occurring substance**						
**Opioid**						
Fentanyl	8 (7.0)	98 (22.6)	<0.001	23 (20.9)	124 (23.7)	NS
Heroin	9 (7.9)	102 (23.5)	<0.001	11 (10.0)	136 (26.0)	<0.001
Methadone	8 (7.0)	14 (3.2)	NS	8 (7.3)	12 (2.3)	<0.05
Morphine	18 (15.8)	162 (37.3)	<0.001	37 (33.6)	170 (32.5)	NS
Other fentanyl analog^†^	<5 (1.8)	112 (25.8)	<0.001	19 (17.3)	99 (18.9)	NS
Oxycodone	11 (9.6)	44 (10.1)	NS	<5 (3.6)	44 (8.4)	NS
**Other**						
Alcohol	37 **(**32.5)	104 (24.0)	NS	33 (30.0)	105 (20.1)	<0.05
Alprazolam	27 (23.7)	84 (19.4)	NS	18 (16.4)	128 (24.5)	NS
Cocaine	58 (50.9)	223 (51.4)	NS	59 (53.6)	218 (41.7)	<0.05
Methamphetamine	10 (8.8)	31 (7.1)	NS	16 (14.5)	37 (7.1)	<0.05

**Abbreviation:** NS = not significant.

* By chi-squared test or Fisher’s exact test when expected cell counts were <5.

^†^ Beginning in January 2016, Florida tested for and reported the following analogs: acetyl fentanyl, beta-hydroxythiofentanyl, butyryl fentanyl/isobutyryl fentanyl, carfentanil, despropionyl fentanyl (4-ANPP), despropionyl fluorofentanyl, fluorobutyryl/fluoroisobutyryl fentanyl, fluorofentanyl, and furanyl fentanyl.

**FIGURE 1:**
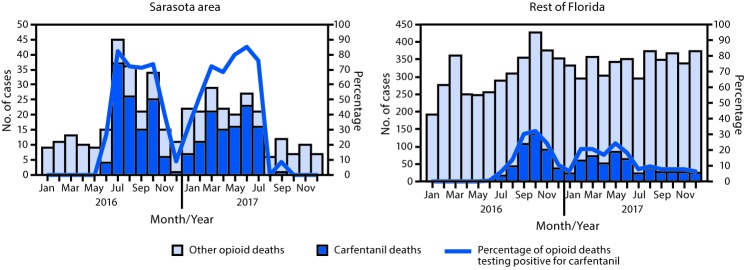
Carfentanil- and opioid-involved deaths and percentage of opioid-involved deaths testing positive for carfentanil — Sarasota area* and the rest of Florida,^†^ 2016 and 2017 * Sarasota, Manatee, and DeSoto counties (the District Twelve Medical Examiner area). ^†^ Excluding Sarasota area.

**FIGURE 2:**
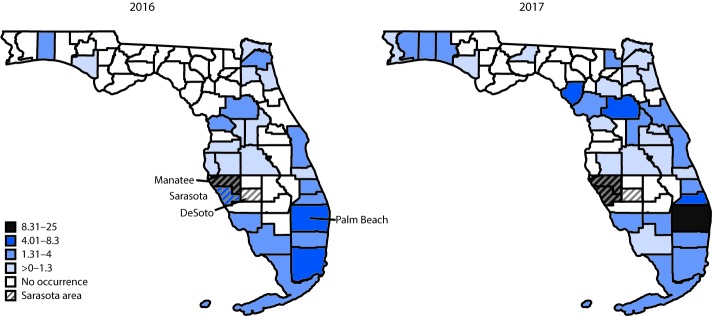
Carfentanil-involved deaths per 100,000 population,* by county where death occurred — Florida, 2016 and 2017^†^ * Most rates are based on counts <20 and should be interpreted with caution. ^†^ Cutpoints for rate categories (based on 2016) determined using Jenks Natural Breaks Optimization in ArcGIS.

During 2016–2017, 35.3% of Sarasota area carfentanil-associated decedents were aged 25–34 years, and the majority were white (89.7%) and male (74.6%) (Table). In 2017, 32% of the carfentanil deaths in the Sarasota area occurred in women, whereas women accounted for 22% of carfentanil deaths in the rest of Florida. During 2016–2017, there was an approximate 59% increase in carfentanil deaths among women (22 versus 35) compared with an approximate 18% decrease among men (92 versus 75). Sarasota area decedents were significantly less likely to have heroin present compared with those in the rest of Florida in both 2016 and 2017, even when including positive morphine results as indicative of a possible heroin death. Carfentanil-involved decedents in the Sarasota area were significantly less likely to have fentanyl or a fentanyl analog other than carfentanil present than were those in the rest of Florida during 2016. In 2017, Sarasota carfentanil deaths became as likely to involve fentanyl or other fentanyl analogs as the rest of Florida (Table). Carfentanil deaths in the Sarasota area in 2017 were significantly more likely to test positive for cocaine (53.6%) and methamphetamine (14.5%) than were those in the rest of Florida (41.7% and 7.1%, respectively); no difference was found for 2016. Differences in cocaine positivity in 2017 were driven by declines in cocaine co-occurrence in the rest of Florida in 2017, whereas differences in methamphetamine positivity were driven by increased co-occurrence in Sarasota area in 2017.

## Discussion

The carfentanil-involved fatal overdose outbreak in the Sarasota area epicenter began in June 2016, lasted for at least 12 months, and abruptly ended in August 2017. By the end of 2017, carfentanil had spread throughout the state and was still present in approximately one in 15 opioid-involved overdose deaths in Florida. Further evidence of carfentanil’s ongoing presence in Florida’s drug supply comes from Palm Beach County, Florida, where carfentanil became the second most frequently detected drug behind alcohol in impaired driving cases during this outbreak (*7*). These findings highlight the need for rapid implementation of geographically targeted and sustained multisector interventions to reduce the proliferation and impact of similar outbreaks as early as possible.

There was significant in-state variation regarding the presence of fentanyl and fentanyl analogs in carfentanil deaths. Earlier in the outbreak, fentanyl and fentanyl analogs were infrequently detected in carfentanil deaths in the Sarasota area, in contrast to those in the rest of Florida. In addition, heroin was coinvolved in nearly 9% of carfentanil deaths in the Sarasota area, compared with approximately 25% of carfentanil deaths in the rest of Florida. This suggests that a substantial percentage of the early carfentanil deaths in Sarasota involved drug products in which the only illicit opioid present was carfentanil. However, as the Sarasota area outbreak spread, fentanyl and other fentanyl analogs were more frequently detected, as was heroin, to a lesser extent. These findings from later in the outbreak are consistent with the pattern of new drug products being mixed with more diverse adulterants over time as they are exchanged by persons involved in the illicit drug market (*5*). Whether decedents were aware of the presence of carfentanil in drug products is unknown.

This is one of the first examinations of a large number of carfentanil fatalities (>1,000 deaths) showing the disproportionate spatiotemporal intensity associated with an outbreak. This outbreak event started at a specific point in time and space, which provided an opportunity to examine changes in drug markets that might have led to these deaths. Law enforcement and the media provided early signals in Florida before mortality data became available, highlighting the need to communicate and share local data across multiple agencies to inform a timely, data-driven response. These findings, along with those from a concurrent carfentanil outbreak in Wayne County, Michigan (*8*), and the timing of carfentanil-positive international drug seizures, suggest that Florida’s outbreak was one of multiple global events leading to the rapid introduction and reduction of carfentanil availability in the illicit drug supply.^§^ In the first half of 2018, the national illicit supply of carfentanil appeared to drop sharply, indicated by an 83% reduction in carfentanil-positive laboratory submissions to the National Forensic Laboratory Information System.^¶^ A similar supply reduction might have occurred in Florida and would partially explain the substantial decline in carfentanil deaths in Florida during late 2017. Closer analyses of law enforcement laboratory submissions and overdose deaths during that period are needed to study this hypothesis.

The findings in this report are subject to at least three limitations. First, medical examiners’ cause of death determinations involve subjective clinical evaluation of incomplete information and different laboratory testing protocols, and thus, might vary across districts in Florida. Second, reports are based on the county where death occurred, which might be different from those where the drug was used, acquired, or where a decedent resided, which might have resulted in misclassification of location. Finally, misclassification of heroin-associated deaths might have occurred: in 55 Sarasota area carfentanil deaths, morphine was detected, but 6-acetylmorphine was absent. Only the presence of 6-acetylmorphine can confirm heroin use; therefore, heroin-involved cases might be undercounted (*9*). Sensitivity analyses was performed by reclassifying these cases as heroin-involved, but the findings did not change.

New data platforms and sharing of disparate data sources (e.g., forensic laboratory samples, dark web advertising, drug-focused social media, emergency medical services transports, and nonfatal overdose mapping) across local agencies might help identify and respond to emergent drug trends more rapidly (*6*,*10*). Routine testing for fentanyl analogs and identifying novel substances in biologic specimens remains a significant national challenge because of the cost and time needed for method validation.**^,^^††^ CDC’s Enhanced State Opioid Overdose Surveillance Program provides funding to 32 states (including Florida) and the District of Columbia, to support comprehensive toxicology testing of opioid overdose decedents, along with rapid overdose surveillance using emergency department data that might help identify emerging threats such as fentanyl analogs.^§§^ In 2019, CDC’s new Overdose Data to Action funding expanded to 49 health departments and included all drug overdose decedents.^¶¶^ With improved testing and surveillance providing actionable intelligence, laboratories, medical examiners, and coroners will be able to alert public health and safety agencies more quickly of suspected overdose outbreaks. This will enable more efficient responses and the deployment of necessary resources to prevent future overdose deaths.

**Summary**

**What is already known about this topic?**

Deaths involving fentanyl analogs, such as carfentanil, have increased the severity of the opioid overdose epidemic in Florida.

**What is added by this report?**

During January 2016–December 2017, 224 of Florida’s 1,181 carfentanil-involved fatal overdoses occurred in the Sarasota area, preceding a larger statewide outbreak in the rest of the state. The outbreak ended in the Sarasota area in August 2017, but carfentanil continued to be detected in overdose deaths in other areas of Florida through the end of 2017.

**What are the implications for public health practice?**

Accelerated reporting, better communication and data sharing across agencies, and increased surveillance for novel substances are needed to mitigate harm associated with introduction of illicitly manufactured fentanyl analogs into drug markets.

## Footnotes

* Fentanyl-related overdoses in Florida before analog testing, during 2013–2015 available at https://www.cdc.gov/mmwr/volumes/65/wr/mm6533a3.htm.

† Annual estimates of the resident population, April 1, 2010 to July 1, 2018 available at https://factfinder.census.gov.

§ https://www.who.int/medicines/access/controlled-substances/Critical_Review_Carfentanil.pdf.

¶ https://www.nflis.deadiversion.usdoj.gov/Resources/NFLISPublicResourceLibrary.aspx.

** Review of fentanyl and fentanyl analogs chemistry available at https://doi.org/10.1016/j.neuropharm.2017.10.016.

†† In 2019, Traceable Opioid Material Kits (TOM Kits) containing traceable opioid reference material were developed by the National Center for Environmental Health at CDC to improve and support laboratory detection of emerging opioids. https://www.cdc.gov/nceh/dls/pdf/Opiod_Factsheet-508.pdf.

§§
https://www.cdc.gov/drugoverdose/foa/state-opioid-mm.html .
